# Concentrations of Glypican-4, Irisin and Total Antioxidant Status in Women with Metabolic Syndrome: Influence of Physical Activity

**DOI:** 10.3390/biom14070768

**Published:** 2024-06-27

**Authors:** Teresa Grzelak, Marcelina Sperling, Marta Pelczyńska, Aniceta Ada Mikulska-Sauermann, Paweł Bogdański, Krystyna Czyżewska, Edyta Mądry

**Affiliations:** 1 Chair and Department of Physiology, Poznan University of Medical Sciences, 6 Święcickiego Street, 60-781 Poznan, Poland; emadry@ump.edu.pl; 2 Department of Medical Chemistry and Laboratory Medicine, Poznan University of Medical Sciences, 8 Rokietnicka Street, 61-701 Poznan, Poland; msperling@ump.edu.pl; 3 Department of Treatment of Obesity, Metabolic Disorders and Clinical Dietetics, Poznan University of Medical Sciences, 84 Szamarzewskiego Street, 60-569 Poznan, Poland; mpelczynska@ump.edu.pl (M.P.); pbogdanski@ump.edu.pl (P.B.); 4 Department of Physical Pharmacy and Pharmacokinetics, Poznan University of Medical Sciences, Rokietnicka 3, 60-806 Poznan, Poland; amikulska@ump.edu.pl; 5 Doctoral School, Poznan University of Medical Sciences, Bukowska 70, 60-812 Poznan, Poland; 6 Department of Nursing, Stanislaw Staszic State University of Applied Sciences in Pila, 10 Podchorążych Street, 64-920 Pila, Poland; kczyzewska@ans.pila.pl

**Keywords:** physical activity, anthropometry, metabolic syndrome, obesity, glypican-4, irisin, antioxidant status

## Abstract

Glypican-4 belongs to a group of poorly understood adipokines, with potential importance in people with metabolic syndrome, especially in groups of patients with glucose metabolism disorder. This study aimed to assess the effect of physical activity on serum glypican-4 and irisin levels and total antioxidant status (TAS) in plasma and saliva in women with metabolic syndrome (MetS). Seventy-two Caucasian women aged 25–60 were included in the study (36 women with MetS and 36 women without MetS (control group, CONTR)). The glypican-4 and irisin concentrations, total antioxidant status, glycemia, lipid profile, anthropometric parameters, and blood pressure were analyzed before and after 28 days of controlled physical activity. Serum glypican-4 and plasma TAS levels were higher (*p* = 0.006 and *p* = 0.043, respectively) on the 28th day than on the first day of the study only in the CONTR group. In the MetS group, 28 days of physical activity caused a reduction in body fat mass (*p* = 0.049) without changes in glypican-4, irisin, or TAS levels. In both groups, glypican-4 levels correlated positively with irisin levels and negatively with Waist-Hip Ratio (WHR), while irisin levels correlated positively with High-Density Lipoprotein Cholesterol (HDL-C) levels and negatively with waist circumference (WC) and WHR values on the 28th day of the study. To summarize, a 28-day moderate training, accompanied by a reduction in body fat mass, stabilized glypican-4 levels and TAS in female patients with MetS.

## 1. Introduction

Glypican-4 belongs to a group of poorly understood membrane heparan sulphate proteoglycans that, under the influence of a specific lipase (insulin-regulated glycosylphosphatidylinositol-specific phospholipase D), can be released into the bloodstream. The gene for the protein is located in chromosome X (Xq26.1; chrX: 133.3–133.42 Mb) [1], and its expression has been noted in many organs, but most notably in the pituitary gland, colon, and adipose tissue. The primary role of glypican-4 is to influence the development and differentiation of cells, including adipocytes, and to affect glucose metabolism [2,3]. Preadipocytes mouse embryonic fibroblast-adipose-like cell line (3T3-L1) with the silenced glypican-4 gene do not undergo differentiation to become mature adipocytes and are characterized by poorer activation of the insulin signaling pathway. However, excessive expression of this gene intensifies insulin-induced phosphorylation of C/EBPb transcription factor (cAMP response element binding protein) and increases the uptake of 2-deoxyglucose [2]. Glypican-4 modifies the canonical and non-canonical wingless/integration signaling (Wnt) [4], which co-ordinates changes in the cellular metabolism that depends, among other things, on the availability of nutrients, such as fats, glucose, or glycogen. Changes in Wnt pathway activity may lead to a disturbance of homeostasis observed in people with obesity, type 2 diabetes, or cardiovascular diseases [5,6,7].

Irisin is classified as an adipokine and myokine [8]. It constitutes a fragment of fibronectin type III domain-containing protein 5 (FNDC5), a protein extracellular domain, which is released under the influence of physical activity or peroxisome proliferator-activated receptor-gamma coactivator 1 alpha—an important molecule that regulates energy metabolism (PGC-1 alpha stimulation). High expression of the FNDC5 gene located on chromosome 1 (1p35.1) has been noticed in adipose and muscle tissue [8,9]. The role of irisin in physiological and pathophysiological processes in the human body needs to be better understood [10,11]. This protein is mainly thought to be important in the metabolism of adipose tissue cells, thermal regulation, and insulin sensitivity. Moreover, irisin is thought to play a role in telomere extension [11,12,13].

In correct conditions, reactive oxygen and nitrogen species in low concentration work primarily as secondary messengers that can regulate intracellular signal transduction pathways. To prevent homeostasis disturbances, excess radicals are eliminated by inter- and extracellular defense mechanisms in which enzymatic and non-enzymatic antioxidants participate [14]. However, an uncontrolled increase in the level of reactive forms of radicals (as a result of, for example, an unhealthy lifestyle or metabolic disease) has a negative effect on the structure and functions of cells on many levels (mainly on nucleic acids, proteins, and unsaturated fatty acids) [15].

The exact reasons for metabolic syndrome and pathophysiological mechanisms related to this condition are not fully known. Broad studies of patients with metabolic syndrome (MetS), i.e., persons who are mainly at high risk of developing dyslipidemia, insulin resistance, and type 2 diabetes, which lead to many chronic illnesses primarily related to the cardiovascular system, increase the chance of early diagnosis and behavioral therapy, as well as dietary treatments that could prevent serious metabolic consequences for those patients [16,17].

To our best knowledge, no report on the association of serum glypican-4 level with physical activity in metabolic syndrome patients, especially in complex to serum concentrations of irisin and total antioxidant status (TAS) in plasma and saliva, exists so far. Thus, this study’s main aim was to assess the effect of physical activity on serum glypican-4 and irisin levels and total antioxidant status (TAS) in plasma and saliva in women with and without metabolic syndrome (MetS). We also analyzed whether physical activity affects the components of MetS (glycemia, lipid profile, and blood pressure) and whether this correlates with glypican-4 and irisin concentration changes.

## 2. Materials and Methods

### 2.1. Characteristics of the Studied Population

**Figure 1:**
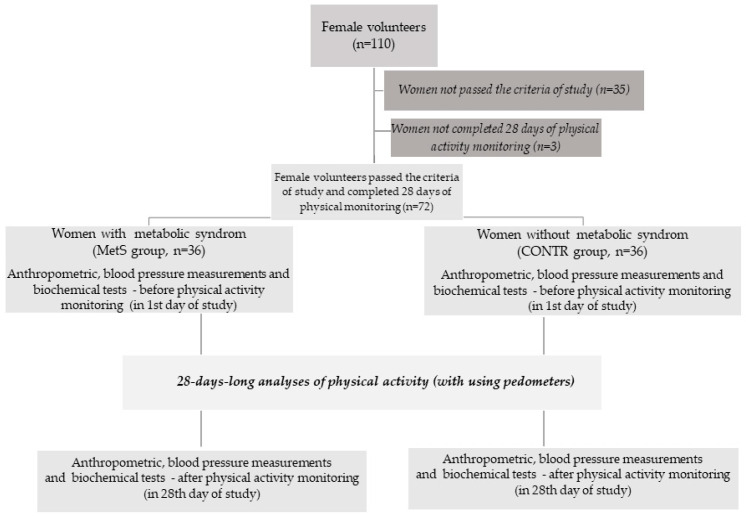
A flowchart with details of the study design.

After physical examination, taking a medical history, initial laboratory tests, anthropometric, and biochemical analyses from the 110 participants initially eligible for the study, seventy-two Caucasian women aged 25–60 were qualified, including 36 women with MetS and 36 women without MetS (control group, CONTR, Figure 1). Inclusion criteria for the MetS group were based on the current guidelines of the International Diabetes Federation [18]. MetS was diagnosed in women who had at least three out of five medical conditions diagnosed: 1. waist circumference (WC) exceeding 80 cm; 2. increased glycaemia after overnight fasting (over 5.6 mmol/L); 3. elevated blood pressure (over 130 mmHg for systolic blood pressure (SBP) and/or over 85 mmHg for diastolic blood pressure (DBP); 4. hypertriglyceridaemia (over 1.7 mmol/L); and 5. decreased High-Density Lipoprotein Cholesterol value (HDL-C, below 1.3 mmol/L). In the MetS group, 100% of women had visceral obesity, 53% had hypertension, 39% had dyslipidemia with a reduced HDL-C value, 28% had hypertriglyceridemia, and 28% had impaired glucose tolerance or type 2 diabetes. Disorders in the CONTR group were rare. All women were without visceral obesity; only 17% of women had hypertension, 3% had reduced HDL-C levels, 3% had high triglycerides levels, and 3% had impaired glucose tolerance.

Exclusion criteria for the MetS and CONTR groups were as follows: genetic syndromes, secondary obesity and hypertension, hepatic, renal, adrenal, and thyroid dysfunction, information provided during medical interviews about diabetes treated with insulin, neoplastic and auto-immunological diseases, alcohol addiction, or symptoms of an acute infection within three months before the analyses. Additionally, there were no patients diagnosed with a limitation that could influence physical activity (congenital or acquired spinal injuries). The analyses did not cover pregnant or lactating women or persons who used medications that affect body weight and composition. Numerous exclusion criteria significantly reduced the size of the study group. In addition, not all were able to complete the entire analysis period (three women dropped out during the 28-day physical activity monitoring and were rejected) because they did not report at the end of the study period, so complete physical and biochemical analyses were impossible. Details of the study design are included in the flowchart in Figure 1.

### 2.2. Physical Activity Analysis

This study was carried out in accordance the Guidelines for Good Clinical Practice (according to the WMA Declaration of Helsinki, Local Bioethics Committee no 729/17 and 326/18). All persons who qualified for the analyses and agreed to participate in the study had been informed about the purpose and the course of the study and were trained on using wristband pedometers (and told to increase their physical activity and walk at least six thousand steps daily).

Physical activity level was checked twofold. All persons who were qualified for the tests had their physical activity level checked over 28 days (number of steps walked, distance covered, and energy used throughout the day) using a specialized device with motion sensors, a data collection program, and considerable memory (Beurer Bluetooth^®^ Activity Tracker AS80 with Health Manager Application, Ulm, Germany). Additionally, on the first and last day of the study, a survey was carried out using the International Physical Activity Questionnaire (IPAQ, using extended versions that cover the previous seven days before the survey), which made it possible to assess, among other things, the level of activity related to walking, intense and moderate physical activity, or total physical activity at work and outside work based on 27 standardized questions [19,20]. All analyses were carried out during the regular, non-vacation season to avoid random changes in physical activity.

### 2.3. Anthropometric and Blood Pressure Measurements

In the MetS and CONTR groups, anthropometric analyses were carried out after overnight fasting twice (at the beginning and the end of the four weeks). The analyses included checking body composition via employing bioelectrical impedance analysis using a TANITA BC-418 device (Tanita Corp., Tokyo, Japan), taking body weight and height measurements using a certified scale with an accuracy of 0.1 kg and a stadiometer (measurement accuracy: 0.1 cm) in light underwear and no shoes, and measuring the circumference of waist, hips, and arms (utilizing a measuring tape). The measurements were then used to calculate classic anthropometric indices, Body Mass Index (BMI) and Waist-Hip Ratio (WHR), and some new ones, Waist-Height Ratio (WHtR) and Body Adiposity Index (BAI—quotient of the circumference of hips and body height, to the power of 1.5; minus 18). After a 10-min rest in a sitting position, the patient’s blood pressure was measured three times as recommended by the European Society of Hypertension and European Society of Cardiology [21] using a sphygmomanometer (model 705IT, Omron Corporation, Kyoto, Japan). All devices used to take measurements had the required certificates.

### 2.4. Biochemical Tests

Samples of venous blood were collected in the morning (7:00–8.00) from every participant after overnight fasting (approximately 12 h after the last meal) and an all-night rest on day 1 (I) and day 28 (II) of the study. Parts of the samples needed to assay glypican-4, irisin, and TAS levels were appropriately secured and frozen at a temperature of −80 °C. Non-stimulated mixed saliva was sampled following the method developed by Fontana et al. [22]. Most of the analyses, i.e., measurements of glucose, triglyceride (TG), total cholesterol (TC), and HDL-C, were carried out immediately using reference enzymatic methods on a cobas e-analyzer (Roche Diagnostic GmbH, Mannheim, Germany). Low-Density Lipoprotein Cholesterol (LDL-C) concentration was calculated using the following formula: [LDL-C = TC − (HDL-C + TG/2.2)], because triglyceridaemia was lower than 4.52 mmol/L.

Evaluation of glypican-4 and irisin concentrations was evaluated in serum using immunoenzymatic tests according to the manufacturer’s instructions (Shanghai Sunredbio (SRB) Technology Co., Ltd., Shanghai, China). The absorbance was read on a MR-96 microplate reader manufactured by CLINDIAG SYSTEMS B.V.B.A. (Pollare, Ninove, Belgium) that made it possible to chart a 4-parameter calibration curve (using Sigma Plot 11.00 software) and determine glypican-4 and irisin levels in serum. The Coefficients of Variation (CVs) intra-assay (CV intra-assay) and inter-assay (CV inter-assay) were, respectively, 5.0% and 6.1% for glypican-4, whereas for irisin they were, respectively, 4.8% and 5.2%.

TAS in plasma and non-stimulated mixed saliva was assayed using a colorimetric analysis with a test by Randox Laboratories Ltd. (Crumlin, Crumlin, UK). Measurements involved incubation of the plasma/saliva with a patented molecule, 2,2′-azino-bis(3-ethylbenzothiazoline-6-sulphonic acid) (ABTS), and then incubation with peroxidase (metmyoglobin) and hydrogen peroxide, which produced ABTS+ radical cations with a characteristic color detected at wavelength 600 nm [using a MR-96 microplate reader by CLINDIAG SYSTEMS B.V.B.A. (Pollare, Ninove, Belgium)]. Level results of the assayed parameters are presented as Trolox equivalents [mmol/L] (water-soluble analog of vitamin E, i.e., 6-hydroxy-2,5,7,8-tetramethylchroman-2-carboxylic acid). The CV intra-assay result for TAS was 4.2% and CV inter-assay result was 5.3% when assayed in plasma. For saliva samples, the CV intra-assay and CV inter-assay were 4.9% and 6.2%, respectively.

### 2.5. Statistical Analyses

The statistical analyses of the results were conducted using STATISTICA 13 software (StatSoft Inc., Tulsa, OK, USA) with Medical Set (in the case of the receiver operating characteristic curves (ROC curves) and the area under the curve (AUC) of the receiver operating characteristic) and IBM SPSS v.25 for Windows (IBM Inc., Armonk, NY, USA). The normality of the distribution of quantitative variables was checked with the Kolmogorov–Smirnov test, and homogeneity of variance was tested using Levene’s test. The results are presented as the means, medians (with interquartile range), and standard deviations (SD). The Wilcoxon matched-pairs signed-rank test was used to compare the levels of measured parameters on the 1st (I) and the 28th day of the study (II) in the case of nonparametric data, separately in the MetS and CONTR groups. The statistical evaluation of differences between the groups was performed using Student’s *t*-test for normally distributed variables, and the nonparametric Mann–Whitney test was used when the distributions were significantly different from normal. A mixed model ANOVA was additionally used to assess the combined influence of both between-subject and within-subject factors after verifying which data in each group met the strict assumptions necessary for the correct application of this statistical analysis. Associations between quantitative variables were characterized by the Pearson correlation coefficient (for parametric data distribution) and Spearman’s rank correlation coefficient (for nonparametric data distribution). The effect of the variables on glypican-4 values was analyzed using logistic regression models. The fit of the logistic regression models was assessed using the likelihood index chi-square test (LRI) and the Hosmer–Lemeshow goodness-of-fit test. The statistically significant level of error was established at α < 0.05.

## 3. Results

Results of analyses of physical activity using pedometers and an IPAQ, anthropometric measurements, and biochemical analyses for specific parameters (irisin and glypican-4 levels in blood serum and TAS in plasma and saliva) obtained at the beginning of the study and the end of the 28 days are presented in Table 1, Table 2 and Table 3. Concerning the CONTR group, the serum concentration of glypican-4 was higher by 13% (*p* = 0.006) on the 28th day of the study than on the first day. Additionally, the level of this adipokine was higher by 12% (*p* = 0.049) when we compared the CONTR vs. MetS groups on the 28th day of the study. In contrast, the irisin concentration in serum and TAS levels in plasma and saliva did not change in all analyzed groups except the CONTR group. Namely, in the CONTR group, we observed an increase of 10% (*p* = 0.043) in plasma TAS level on the 28th day of the study in comparison to the first day.

After verifying the mixed effect of the two factors, time (before vs. after 28 days of physical activity monitoring) and group (CONTR vs. MetS) in mixed model ANOVA, the results showed that there was no interaction effect between these two factors. The result of the main effect of such a factor as the kind of group was appropriate, with statistical significance (*p* < 0.0001) only for HDL-C levels (higher concentrations in CONTR vs. MetS). We also found no effect of the abovementioned factors on the obtained total cholesterol level (Table 4).

Moreover, about a 3% loss of fat body mass value (measured in kg, *p* = 0.049) and a positive correlation between the concentration of irisin (II) and the level of physical activity related to walking based on the IPAQ (R = 0.29, *p* = 0.044) were noticed in MetS group. In the CONTR group, we observed a small but statistically significant increase in the case of arm circumference values (*p* = 0.011) and a decrease in DBP (*p* = 0.029) and SBP (*p* = 0.033).

After taking into account adjustments for glypican-4 level concerning fat body mass (%), it was noted that the Glypican-4/Fat Body Mass (%) ratio was higher at the end of the 28-day analysis period compared to the initial value obtained in the MetS (*p* = 0.004) and CONTR (*p* < 0.0001) groups. In contrast, in the case of a similar adjustment for irisin levels [Irisin/Fat Body Mass (%)], the increase concerned only the CONTR group (*p* < 0.0001). The level of the Glypican-4/Fat Body Mass (%) ratio at the beginning (*p* = 0.033) and at the end of the physical activity analyses (*p* < 0.0001) was lower in the MetS vs CONTR group (Table 3). Similar results were obtained for the Irisin/Fat Body Mass (%) ratio (*p* = 0.0024 and *p* < 0.0001, respectively).

The number of steps walked (according to the pedometers) in women with MetS showed a positive correlation with total physical activity level according to the IPAQ (R = 0.37), as well as with the level of physical activity related to walking according to the IPAQ (R = 0.36) and with the percentage of lean body mass (R = 0.48). There was also a negative correlation between the number of steps walked and glycaemia (II) (R = −0.38), non-HDL-C level (II) (R = −0.34), DBP (II) (R = −0.37), BMI (II) (R = −0.36), and percentage of body fat (II) (R = −0.47). Such correlations were not noted in the control group.

Similar correlations to the ones presented above were noted in the MetS group between the period of physical activity duration during the day, according to the pedometers, and selected conditions related to metabolic syndrome (WC, BMI, DBP, and glycaemia), as well as the percentage of fat adipose tissue and non-HDL-C level. Detailed data referring to correlation and dependence are presented in Table 5. On the other hand, in the CONTR group, there was no correlation between the duration of physical activity and the medical conditions, which are characterized by the diagnosis of metabolic syndrome.

In the MetS group, in the case of the glypican-4 (II) level, a positive correlation was observed with the irisin level (II) (R = 0.64) and glycaemia (II) (R = 0.33). In the CONTR group, at the end of the 28 days, there was a positive correlation between the glypican-4 (II) level and the level of irisin (II) (R = 0.86), whereas a negative correlation was noted with the value of the WHR (II) (R = −0.36).

**Figure 2:**
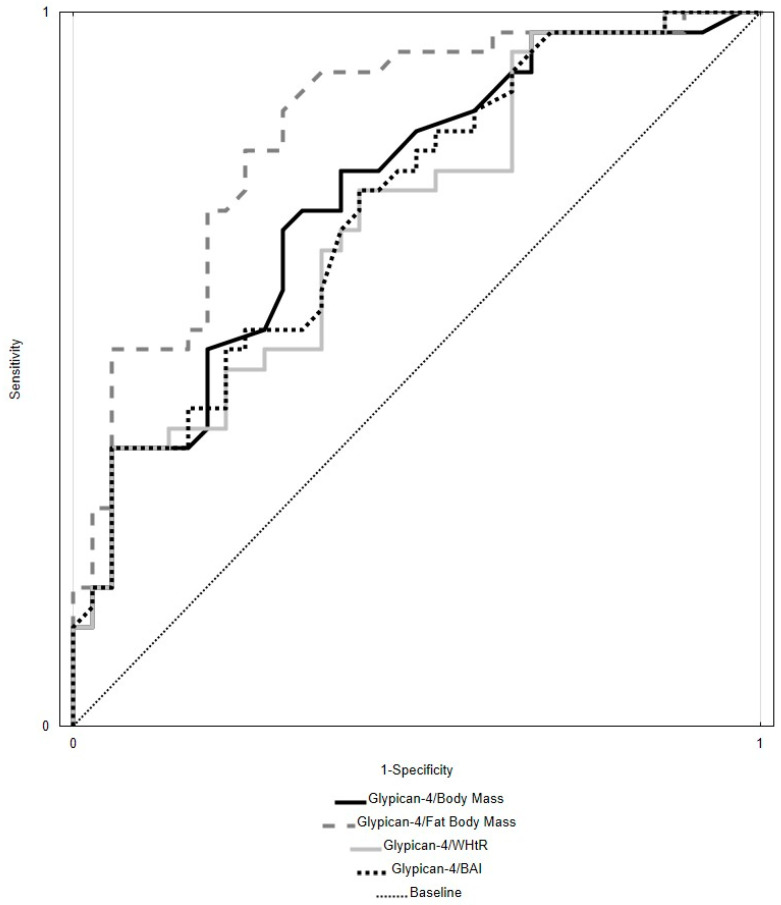
Receiver operating characteristic curves for indexes: Glypican-4/Body Mass, Glypican-4/Fat Body Mass (%), Glypican-4/WHtR, and Glypican-4/BAI upon a comparison between MetS (metabolic syndrome) and CONTR (control) groups in the 28th day of analyses. WHtR—Waist–Height Ratio; BAI—Body Adiposity Index.

The comparative analysis of ROC curves (presented in Figure 2) revealed that the Glypican-4/Fat Body Mass (%) ratio is a good predictor of metabolic disorders (with an 82% specificity level at the cut-off point being 70.00 pg/mL/%). Limited but still satisfactory usefulness was established for the following ratios: Glypican-4/Body Mass (AUC = 0.744), Glypican-4/BAI (AUC = 0.726), and Glypican-4/WHtR (AUC = 0.710), whereas low AUC curve values were noted for the Glypican-4/BMI, Glypican-4/WHR, and Glypican-4/Fat-Free Body Mass (%) ratios (Table 6, Figure 2).

**Figure 3:**
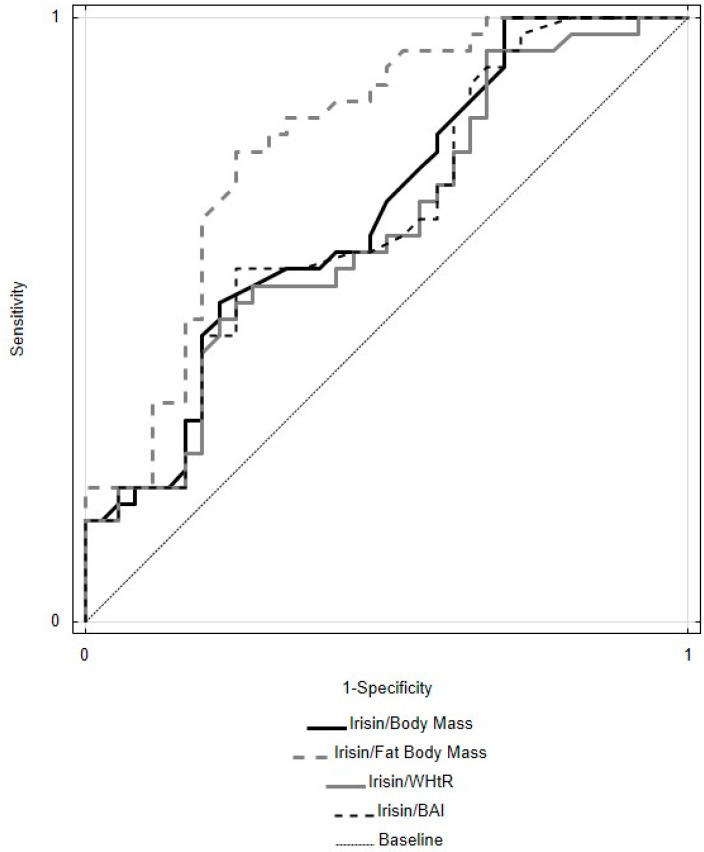
Receiver operating characteristic curves for indexes: Irisin/Body Mass, Irisin/Fat Body Mass (%), Irisin/WHtR, and Irisin/BAI upon a comparison between MetS (metabolic syndrome) and CONTR (control) groups in the 28th day of analyses. WHtR—Waist–Height Ratio; BAI—Body Adiposity Index.

In the MetS group, irisin (II) concentration was positively correlated with TG (II) level and DBP (II) value (R = 0.46 and R = 0.38, respectively). In the CONTR group, the irisin (II) profile, apart from the positive correlation with glypican-4 (II) concentration (R = 0.86), had negative correlations with TC (II) (R = −0.38), LDL-C (II) (R = −0.39), and non-HDL-C (II) (R = −0.38). Similarly to the Glypican-4/Fat Body Mass (%) ratio, comparative analysis of ROC curves revealed that the AUC value for the Irisin/Fat Body Mass (%) ratio as a predictor for metabolic syndrome is statistically significantly different from analogous parameters for the following ratios: Irisin/Body Mass, Irisin/BMI, Irisin/Fat-Free Body Mass (%), Irisin/WHR, Irisin/WHtR, and Irisin/BAI (Table 7, Figure 3).

TAS levels in plasma were higher than in the saliva, both in the case of the MetS group (*p* = 0.030 on the first day of the analyses and *p* = 0.040 on the last day) and CONTR group (similarly, *p* = 0.004 and *p* < 0.0001), but if we compare groups of women with MetS and those without MetS, as well as the status before and after 28 days of controlled physical activity, then the profile of this parameter did not change significantly from the statistical point of view. However, positive correlations were observed in the MetS group concerning TAS (II) levels in plasma vs. TAS (II) in saliva (R = 0.38). Additionally, according to the IPAQ, a positive correlation existed between TAS (II) in saliva and total physical activity (R = 0.36) and physical activity related to walking (R = 0.45). As regards the CONTR group, a positive correlation was noted concerning TAS (II) level in plasma and TAS (II) level in saliva (R = 0.42).

According to the logistic regression model, in the MetS group, each one-unit increase in TAS levels in plasma (presented in mmol/L) at the end of the 28-day physical activity increased by 58.6 times the chance to obtain a lower glypican-4 level at the end of the study (LRI = −18.465, *p* = 0.007) in comparison to the values of this adipokine level obtained at the beginning of the analysis. The Hosmer–Lemeshow statistic was 8.420 (*p* = 0.394); therefore, the model was judged to fit the observed data well [23]. In this group, the value of R^2^ Nagelkerke was equal to 0.262. In the CONTR group, the influence of TAS level in plasma on serum glypican-4 level was not statistically significant (Table 8).

## 4. Discussion

The importance of moderate, regular physical activity in preventing obesity, insulin resistance, dyslipidemia, and metabolic syndrome is widely accepted, but the mechanism itself is poorly understood [16,17]. This study has shown that the higher the number of steps and time devoted to physical activity, the more positively it translates into anthropometric measurements in the case of women with MetS. This study’s results, which included the use of the IPAQ, were similar to those taken from pedometers; it is worth considering both, but note that in the case of the pedometer, the results relate to walking only [21]. Our results obtained, among other analyses, during mixed model ANOVA showed no specific effect of moderate activity (at a mean of seven thousand steps a day) during four weeks on the TC and HDL-C levels in both diagnosed groups. Most MetS patients were using lipid monitoring drugs, which may have affected TC and HDL-C values more than physical activity levels. Moreover, another factor (for example, diet) may have influenced the lipid profiles in the CONTR and MetS groups [17].

Physical activity did not influence the level of glypican-4 in women in the MetS group, similar to in the study by Yoo et al. [24], which showed that a 3-month combined aerobic and resistance exercise program did not change circulating glypican-4 levels in obese women. Our results also followed other authors’ findings, which showed endurance training (14 weeks of moderate-intensity treadmill running) did not cause a change in serum level of glypican-4 in streptozotocin-nicotinamide-induced diabetic male rats [25], and in the case of resistance training (12 weeks of male rats climbing on a ladder with a weight hanging from the rat’s tail in streptozotocin-induced diabetic animals) [26]. Because, in the MetS group, there were both women with prediabetic stage and female patients with type 2 diabetes, it is possible the stabilized level of this adipokine in serum was connected with the fact observed by Ussar et al. [3] that, in prediabetic subjects, the glypican-4 level is elevated, but in patients with type 2 diabetes it is decreased, in comparison with healthy persons.

In our CONTR group (with correct WHR values), the adipokine level was higher on the 28th day of increased physical activity compared to the first day of the study. The selective impact of physical activity on glypican-4 concentration levels was also noted in research conducted by Yoo et al. [24] in 40 nondiabetic obese women with nonalcoholic fatty liver disease. Yoo et al. [24] observed no discernible change in concentration of this adipokine in plasma after a 3-month mixed training program (aerobic and resistance), but plasma glypican-4 levels in subjects with a reduced WHR after physical activity showed a tendency to decrease, whereas those with an increased WHR after the training program showed a tendency to increase in adipokine level. As is well known, adipose tissue and skeletal muscle contribute to systemic levels of glypican-4 [3], which may partly explain the increase in serum levels of this adipokine we noted only in the group of women without MetS, as we observed a loss of body fat mass after 28 days of physical activity in our MetS group, but not in the CONTR group, especially since only in the CONTR group did we observe an increase in arm circumference at the end of the study.

Our analyses confirmed positive correlations between glypican-4 and irisin levels in serum in both studied groups (MetS and CONTR). However, such a correlation was more pronounced in the group of women without the characteristics of MetS. Since there are no similar analyses (in the studies of other authors) on this correlation, it can only be assumed that the activity of both molecules is at the level of a peroxisome proliferator-activated-γ-receptor (PPAR-γ). It has been shown that activation of this receptor increases the mRNA level for glypican-4 and the expression of this protein in subcutaneous fatty tissue in mice [27], whereas the administration of an activator for the PPAR-γ receptor (peroxisome proliferator-activated receptor-gamma coactivator 1 alpha, PGC-1α) in the case of rodents would release irisin from its precursor, similarly to physical activity [8].

Due to the secretion of glypican-4 and irisin by fatty adipose tissue cells, it can be assumed that their concentration is related to anthropometric measurements. Hence, the presented study investigated glypican-4 and irisin concentration levels concerning body mass, BMI, fat body mass (%), fat-free body mass (%), WHR, WHtR, and BAI. The highest predictive value for metabolic disorders was shown by the Glypican-4/Fat Body Mass (%) (AUC = 0.837) ratio, which had high sensitivity and specificity, with the cut-off point being 71.00 pg/mL/%. Most of the recognized diagnostic markers used in clinical studies are characterized by an AUC value according to the ROC curve between 0.8–0.9; with values that are 0.7 and above, the predictor is deemed satisfactory [28].

Cluster analyses revealed that glypican-4 is significant in glucose and lipid metabolism [29]. This adipokine is unique as it acts as a direct stimulator of insulin receptors. In a prediabetic state, its level increases, but in patients with type 2 diabetes, it is lower than in healthy persons [3]. A positive correlation between glypican-4 levels in serum with glycaemia was established in our research (concerning persons with MetS and without the characteristics of this condition) and elevated the circulating glypican-4 level in prediabetic subjects (in the study of Sakkane et al. [4]), suggesting that this proteoglycan affects the modification of carbohydrate metabolism. Adverse changes in glycaemia influencing metabolic control were characteristic of diabetic patients with low glypican-4 values in serum [30]. Moreover, there was a specific relationship between glycaemia and glypican-4 level observed by Ussar et al. [2] in an animal study: random-fed blood glucose and insulin measurements revealed that high-fat diet-fed mice were still able to maintain normal glycemia and normal insulinemia, with much higher serum glypican-4 levels than control animals (with a standard diet), but markedly obese (*ob/ob*) mice had hyperglycaemia despite hyperinsulinemia, which was accompanied with reduced serum glypican-4 levels. Interestingly, we found for the first time that if there was a decrease in glypican-4 levels after 28 days of physical activity in some of the women in the metabolic syndrome group, this was many times more often in the case of female patients characterized by a high value of total plasma antioxidant status at the end of the 4-week study.

Studies on glypican-4 gene expression in slim persons have revealed that the expression is five times more intense in subcutaneous fatty adipose tissue than visceral fatty tissue. As the values of BMI and WHR in overweight persons increase, the expression of glypican-4 in subcutaneous fatty adipose tissue significantly decreases, becoming stable in obese patients, whereas in visceral tissue, it gradually increases [31]. Our research on a group of women with MetS showed a negative correlation between the level of glypican-4 and WHR value, confirming that visceral fatty tissue has a crucial influence on the concentration of this adipokine in serum.

The presented analyses show that irisin concentration in serum did not change for women with MetS or in the CONTR group (comparing results from before the commencement of the study and after four weeks). According to the results of other authors, physical activity increases [8,32] or does not influence [33,34,35,36] the level of this protein in the blood, and discrepancies can be attributed to, among other things, the type, duration, and level of physical activity, as well as to the thermogenic process taking place during exercises [33,34,35]. A meta-analysis showed that acute exercise is associated with a significant post-exercise increase in irisin concentration (in the case of immediately measuring post-exercise). It was connected with the relatively short-term existence of this adipokine in plasma [37]. Moreover, the most recent systematic review and meta-analysis research suggested that only long-term resistance training (but not long-term aerobic training, short-term aerobic training, or short + long-term aerobic training) can elevate irisin levels in serum [38].

After taking into account adjustments of irisin concentration levels compared to the level of Homeostasis Model Assessment for Insulin Resistance (HOMA-IR), it was established that the highest values for the Irisin/HOMA-IR ratio were observed in international and local class athletes (who had low body fat mass content), whereas in people who engage in recreational physical activities (no more than 3 h per week) the ratio was approximately 50% lower. The lowest value was observed in the population with a sedentary lifestyle and relatively high body fat mass content [34]. Taking into account the above, an analogy could be drawn to our analyses in which there are differences in Irisin/Fat Body Mass (%) ratio between the MetS and CONTR groups, and in the control group, differences can also be observed at the beginning and the end of the study.

Higher AUC values for ROC curves were observed for the Irisin/Fat Body Mass (%) ratio vs. Irisin/Fat-Free Body Mass (%). Secretion of irisin takes place both via fatty tissue and muscle tissue. However, studies conducted on laboratory animals have shown that the expression of the FNDC5 gene in adipocytes, but not in myocytes, correlates with the irisin concentration level in the blood of mice which are obese due to a high-fat diet [39]. Because studies show both a positive [27,32,40] and negative [41,42] correlation between irisin level in blood and BMI values, as well as no correlation in our research and research conducted by other authors [10,11], a hypothesis was put forward that concerned an adaptive response involving resistance to irisin in patients with visceral obesity, similar to better-researched resistance to other adipokines (e.g., leptin) or hormones (e.g., insulin) [43].

The values of TAS in saliva increased together with the total level of physical activity according to the IPAQ and with the level of physical activity related to walking according to the IPAQ. Moreover, we observed in the CONTR group, but not in women with MetS, higher values of TAS level in plasma after 28 days of physical activity, compared with the first day of study. Research conducted on persons who engage in sports activities shows an increase in TAS in blood and an increase in antioxidant status in saliva after physical exercises [44,45].

Changes in TAS levels were noticed in the plasma and saliva tests of anorexic patients (compared to their healthy peers), thus in a population with highly varied body weight and composition, as well as physical activity level and supply of antioxidants with diet [46]. Adding nutrients with antioxidant properties to the diet is particularly important to maintain a favorable pro-oxidant–antioxidant balance for sportspeople [47]. It was demonstrated that groups on a well-balanced diet had higher levels of TAS in plasma than those on a non-balanced diet [48]. However, there were no significant changes in this parameter in the case of administration over 90 days of 4 mg/24 h of carotenoids with antioxidant properties (astaxanthin) to soccer players who trained intensively, and a decrease in total oxidant status (TOS) in plasma was noticed [49]. One-time administration of another antioxidant—250 mg of vitamin C—increased the TAS level in the saliva of healthy persons [50].

Oxidant–antioxidant processes concerning persons with MetS and engaged in physical activity are complex. Disturbances in carbohydrate metabolism and/or dyslipidemia and intense exercise promote the production of considerable amounts of free radicals as antioxidants in the system are depleted, which can generate oxidative stress and metabolic complications [47]. The parameter that we applied in our studies—TAS—is a parameter that takes into account the dynamics of changes in the antioxidant capacity of plasma or other body fluids (e.g., saliva) and involves antioxidative enzymes and low-molecular antioxidants. Its assessment helps better describe the antioxidative characteristics of complex biological systems than the sum of concentrations/activity of all antioxidants measured individually [51]. Collecting saliva is one of the few non-invasive medical procedures (contrary to collecting blood samples, etc.). Hence, it is worth using the above for analyses to provide additional information concerning studies on human populations [16,52,53], particularly given that recent research has revealed the presence of a few hundred metabolites in human saliva [52,53].

The presented study has certain limitations, mainly concerning sex and sample sizes. Because it was found before that glypican-4 levels exhibit sex differences (those in men being significantly higher than those in females) [24], we have analyzed only a female population (to avoid a wide dispersion of results). Moreover, we had a social problem with recruiting men for study (many more women than men wanted to be volunteers in our project). Research was carried out over 28 days, studying 36 women with MetS and 36 control women, so the number of participants in the groups was higher than the statistically required minimum; this study was conducted based on pilot studies’ results and with the assumption that the test’s strength is 0.9 and type I error 0.05 (parameters often used in medical studies). There are plans to include a larger population in a future study and divide it into sub-groups according to varied workout intensity to verify the obtained results, particularly regarding a significant dispersion of results. Such a study would also involve checking irisin and glypican-4 levels in saliva.

## 5. Conclusions

A 28-day moderate training period, which was accompanied by a statistically significant reduction in body fat mass, stabilized glypican-4 and total antioxidant status in women with metabolic syndrome. The specific relationships between changes in glypican-4 level in serum and changes in total antioxidant status in plasma in the control group, as well as the high correlation between levels of glypican-4 and irisin in the serum of the female population, suggest that there is a link between the effects of these parameters, but further research is needed to understand this phenomenon.

## Figures and Tables

**Table 1:** Analyses of anthropometry in the study (MetS, n = 36) and control (CONTR, n = 36) groups.

Parameters [Unit]	Median Values ± QD in 1st Day/in 28th Day of Analyses in MetS	Median Values ± QD in 1st Day/in 28th Day of Analyses in CONTR	*p*
Body mass [kg]	84.40 ± 5.62/82.60 ± 5.92	63.20 ± 4.40/63.50 ± 3.75	<0.0001 * <0.0001 ** NS # NS ##
Height [m]	1.65 ± 0.04 #	1.68 ± 0.04 #	NS ***
BMI [kg/m^2^]	30.55 ± 1.71/30.00 ± 2.16	22.30 ± 1.49/22.45 ± 1.55	<0.0001 * <0.0001 ** NS # NS ##
WC [cm]	96.00 ± 6.25/96.00 ± 6.00	76.50 ± 6.00/77.00 ± 6.00	<0.0001 * <0.0001 ** NS # NS ##
Hip circumference [cm]	114.00 ± 5.12/114.00 ± 3.75	98.50 ± 4.25/100.00 ± 4.00	<0.0001 * <0.0001 ** NS # NS ##
Arm circumference [cm]	32.00 ± 2.50/34.00 ± 2.12	26.65 ± 1.75/27.36 ± 0.87	<0.0001 * <0.0001 ** NS # 0.011 ##
Fat body mass [kg]	32.85 ± 4.20/31.95 ± 4.55	17.10 ± 3.10/17.55 ± 3.46	<0.0001 * <0.0001 ** 0.049 # NS ##
Fat-free body mass [%]	51.35 ± 2.36/50.75 ± 2.25	46.50 ± 3.09/46.50 ± 2.70	<0.0001 * <0.0001 ** NS # NS ##
WHR	0.85 ± 0.03/0.84 ± 0.03	0.77 ± 0.04/0.78 ± 0.03	<0.0001 * <0.0001 ** NS # NS ##
WHtR	0.58 ± 0.03/0.58 ± 0.03	0.46 ± 0.03/0.46 ± 0.04	<0.0001 * <0.0001 ** NS # NS ##
BAI	34.85 ± 2.54/35.09 ± 2.39	26.99 ± 1.85/27.97 ± 1.67	<0.0001 * <0.0001 ** NS # NS ##

n—number of people; QD—quartile deviation; BMI—Body Mass Index; WC—waist circumference; WHR—Waist–Hip Ratio; WHtR—Waist–Height Ratio; BAI—Body Adiposity Index; NS—statistically insignificant difference; *p*—level of statistical significance: * for comparison MetS vs. CONTR groups (1st day); ** for comparison MetS vs. CONTR groups (28th day); # for comparison between 1st day and 28th day in MetS group; ## for comparison between 1st day and 28th day in CONTR group; *** levels of statistical significance upon a comparison between MetS group vs. CONTR group in the case of date of 28-day-long analyses of physical activity (using pedometers).

**Table 2:** Analyses of physical activity and blood pressure in the study (MetS, n = 36) and control (CONTR, n = 36) groups.

Parameters [Unit]	Median Values ± QD in 1st Day/in 28th Day of Analyses in MetS	Median Values ± QD in 1st Day/in 28th Day of Analyses in CONTR	*p*
SBP [mm/Hg]	128.50 ± 11.12/129.50 ± 8.87	116.00 ± 7.25/113.50 ± 7.75	0.0003 <0.0001 ** NS # 0.033 ##
DBP [mm/Hg]	79.50 ± 5.75/81.00 ± 7.87	71.00 ± 4.87/69.00 ± 6.12	0.0009 * <0.0001 ** NS # 0.029 ##
Number of steps/24 h (pedometers)	6761.79 ± 1399.32 †	7100.44 ± 2090.23 †	NS ***
Distance/24 h [km] (pedometers)	4.73 ± 1.37 †	4.89 ± 1.34 †	NS ***
Time of physical activity/24 h [min] (pedometers)	55.62 ± 13.22 †	58.17 ± 18.94 †	NS ***
Degree of minimum †† physical activity/24 h [%] (pedometers)	112.17 ± 23.30 †	117.85 ± 31.73 †	NS ***

n—number of people; SBP—systolic blood pressure; DBP—diastolic blood pressure; QD—quartile deviation; † mean ± standard deviations in 28-day-long analyses of physical activity (with using pedometers); †† minimum of physical activity/24 h on the level 6000 steps/24 h; NS—statistically insignificant difference; *p*—level of statistical significance: * for comparison MetS vs. CONTR groups (1st day); ** for comparison of MetS vs CONTR groups (28th day); # for comparison between 1st day and 28th day in MetS group; ## for comparison between 1st day and 28th day in CONTR group; *** levels of statistical significance upon comparison between MetS group vs CONTR group in the case of date of 28-day-long analyses of physical activity (with using pedometers).

**Table 3:** Biochemical analyses in the study (MetS, n = 36) and the control (CONTR, n = 36) groups.

Parameters [Unit]	Median Values ± QD in 1st Day/in 28th Day of Analyses in MetS	Median Values ± QD in 1st Day/in 28th Day of Analyses in CONTR	*p*
TC [mmol/L]	4.85 ± 0.44/4.75 ± 0.53	4.84 ± 0.78/4.87 ± 0.91	NS * NS ** NS # NS ##
LDL-C [mmol/L]	2.89 ± 0.40/2.86 ± 0.44	2.90 ± 0.74/2.74 ± 0.79	NS * NS ** NS # NS ##
HDL-C [mmol/L]	1.45 ± 0.25/1.39 ± 0.26	1.87 ± 0.31/1.86 ± 0.25	<0.0001 * <0.0001 ** NS # NS ##
TG [mmol/L]	1.14 ± 0.46/1.19 ± 0.41	0.79 ± 0.20/0.78 ± 0.22	<0.0001 * 0.0003 ** NS # NS ##
Glycemia [mmol/L]	5.33 ± 0.31/5.34 ± 0.37	4.89 ± 0.23/4.86 ± 0.28	<0.0001 * <0.0001 ** NS # NS ##
Glypican-4 [ng/mL]	1.49 ± 1.02/1.51 ± 0.49	1.50 ± 1.13/1.69 ± 1.38	NS * 0.049 ** NS # 0.0006 ##
Glypican-4/Fat Body Mass [ng/mL/%]	0.037 ± 0.024/0.047 ± 0.021	0.053 ± 0.038/0.128 ± 0.083	0.033 * <0.0001 ** 0.004 # <0.0001 ##
Irisin [ng/mL]	2.19 ± 0.79/1.95 ± 0.72	2.15 ± 2.07/2.00 ± 1.57	NS * NS ** NS # NS ##
Irisin/Fat Body Mass [ng/mL/%]	0.053 ± 0.021/0.051 ± 0.021	0.096 ± 0.087/0.138 ± 0.092	0.0024 * <0.0001 ** NS # <0.0001 ##
TAS [mmol/L] in plasma	1.40 ± 0.16/1.29 ± 0.18	1.32 ± 0.10/1.36 ± 0.11	NS * NS ** NS # 0.043 ##
TAS [mmol/L] in saliva	1.19 ± 0.48/1.12 ± 0.50	0.79 ± 0.58/0.80 ± 0.29	NS * NS ** NS # NS ##

n—number of people; TC—total cholesterol; LDL-C—Low-Density Lipoprotein Cholesterol; HDL-C—High-Density Lipoprotein Cholesterol; TG—triglyceridaemia; TAS—total antioxidant status; QD—quartile deviation; NS—statistically insignificant difference; *p*—level of statistical significance: * for comparison MetS vs. CONTR groups (1st day); ** for comparison MetS vs. CONTR groups (28th day); # for comparison between 1st day and 28th day in MetS group; ## for comparison between 1st day and 28th day in CONTR group.

**Table 4:** Mixed model ANOVA results for levels of TC [mmol/l] and HDL-C [mmol/l] in four subgroups (MetS (n = 36): before and after 28 days of physical activity analyses; CONTR (n = 36): before and after 28 days of physical activity analyses; MetS (n = 36).

Parameters [Unit]	Sum of Squares	df	Mean Squares	F Value	*p*
	**TC [mmol/L]**				
GROUPS (MetS/CONTR)	0.532	1	0.532	0.339	0.562
Error	109.835	70	1.569		
TIME (1st day/28th day)	0.017	1	0.017	0.091	0.763
TIME × GROUPS	0.294	1	0.294	1.609	0.209
Error	12.802	70	0.183		
	**HDL-C [mmol/L]**				
GROUPS (MetS/CONTR)	11,236.0	1	11,236.0	30.533	<0.0001
Error	25,759.6	70	368.0		
TIME (1st day/28th day)	49.0	1	49.0	1.790	0.185
TIME × GROUPS	6.2	1	6.2	0.228	0.634
Error	1915.8	70	27.4		

n—number of people; *p*—level of statistical significance; TC—total cholesterol; HDL-C—High-Density Lipoprotein Cholesterol.

**Table 5:** Indices of correlation and levels of statistical significance in cases of analysis involving the relationship between time of physical activity/24 h according to pedometers and selected anthropometric, and biochemical parameters in 28 days of analyses in a group with metabolic syndrome (MetS).

Parameters [Unit]	Time of Physical Activity According to Pedometers [Min/24 h]
Body weight [kg]	R = −0.33; *p* = 0.049
WC [cm]	R = −0.34; *p* = 0.039
Body fat mass [%]	R = −0.53; *p* = 0.0007
BMI [kg/m^2^]	R = −0.40; *p* = 0.001
DBP [mmHg]	R = −0.36; *p* = 0.030
Physical activity connecting with walking (according to IPAQ) [MET—min/week]	R = 0.36; *p* = 0.029
TC [mmol/L]	R = −0.33; *p* = 0.049
Non-HDL-C [mmol/L]	R = −0.35; *p* = 0.038
Glycemia [mmol/L]	R = −0.42; *p* = 0.010

WC—waist circumference; BMI—Body Mass Index; DBP—diastolic blood pressure; IPAQ—International Physical Activity Questionnaire; TC—total cholesterol; HDL-C—High-Density Lipoprotein Cholesterol; R—coefficient of Pearson or Spearman (for, respectively, parametric or nonparametric data distribution); MET—Metabolic Equivalent of Task; *p*—level of statistical significance.

**Table 6:** Characteristics of receiver operating characteristic (ROC) curves for indexes Glypican-4/Body Mass, Glypican-4/BMI, Glypican-4/Fat Body Mass (%), Glypican-4/Fat-Free Body Mass (%), Glypican-4/WHR, Glypican-4/WHtR, and Glypican-4/BAI for pairs of studied groups (MetS; n = 36 vs CONTR; n = 36) in the 28th day of analyses.

Parameters [Unit]	Cut-Off Value	AUC	SD (AUC)	95% CI	*p*
Glypican-4/Body Mass [ng/mL/kg]	0.022	0.744	0.058	0.631–0.857	<0.0001
Glypican-4/BMI [ng/mL/kg/m^2^]	0.092	0.646	0.065	0.519–0.773	0.024
Glypican-4/Fat Body Mass (%) [ng/mL/%]	0.071	0.837	0.047	0.744–0.930	<0.0001
Glypican-4/Fat-Free Body Mass (%) [ng/mL/%]	0.196	0.686	0.062	0.565–0.808	0.027
Glypican-4 /WHR [ng/mL]	4.245	0.653	0.064	0.527–0.780	0.017
Glypican-4/WHtR [ng/mL]	3.018	0.710	0.060	0.592–0.828	0.0005
Glypican-4/BAI [ng/mL]	0.050	0.726	0.059	0.611–0.842	0.0001

MetS—group with metabolic syndrome; CONTR—control group; n—number of people; cut-off value in ROC (receiver operating characteristic) curve, AUC—area under curve of receiver operating characteristic); SD (AUC)—standard deviation of AUC; BMI—Body Mass Index; WHR—Waist–Hip Ratio; WHtR—Waist–Height Ratio; BAI—Body Adiposity Index; *p*—level of statistical significance.

**Table 7:** Characteristics of receiver operating characteristic (ROC) curves for indexes: Irisin/Body Mass, Irisin/BMI, Irisin/Fat Body Mass (%), Irisin/Fat-Free Body Mass (%), Irisin/WHR, Irisin/WHtR, and Irisin/BAI for pairs of studied groups (MetS; n = 36 vs. CONTR; n = 36) in the 28th day of analyses.

Parameters [Unit]	Cut-Off Value	AUC	SD (AUC)	95% CI	*p*
Irisin/Body Mass [ng/mL/kg]	0.029	0.682	0.062	0.560–0.805	0.003
Irisin/BMI [ng/mL/kg/m^2^]	0.079	0.595	0.067	0.463–0.727	NS
Irisin/Fat Body Mass (%) [ng/mL/%]	0.076	0.796	0.053	0.692–0.900	<0.0001
Irisin/Fat-Free Body Mass (%) [ng/mL/%]	0.159	0.485	0.07	0.347–0.622	NS
Irisin/WHR [ng/mL]	3.983	0.604	0.067	0.473–0.736	NS
Irisin/WHtR [ng/mL]	4.134	0.653	0.065	0.526–0.779	0.018
Irisin/BAI [ng/mL]	0.062	0.669	0.064	0.545–0.794	0.008

MetS—group with metabolic syndrome; CONTR—control group; n—number of people; cut-off value—value on ROC (receiver operating characteristic) curve, AUC—area under curve of receiver operating characteristic); SD (AUC)—standard deviation of AUC; BMI—Body Mass Index; WHR—Waist–Hip Ratio; WHtR—Waist–Height Ratio; BAI—Body Adiposity Index; *p*—level of statistical significance; NS—statistically insignificant difference.

**Table 8:** Factors associated with increased the chance of lower glypican-4 level at the end of the study, compared to the values of this adipokine level obtained at the beginning of the analysis, in the study (MetS) and control (CONTR) groups.

Factors	b	SE (b)	Wald Statistics	OR (95% CI)	*p*
MetS group (n = 36) R^2^ Nagelkerke = 0.262; LRI = −18.465 (*p* = 0.007); the Hosmer–Lemeshow statistic = 8.420 (*p* = 0.394)					
Constant (y-intercept)	−6.288	2.376	7.002	0.002 (0.000–0.196)	0.008
TAS [mmol/L] in plasma	4.071	1.701	5.727	58.648 (2.089–1646.187)	0.017
CONTR group (n = 36) R^2^ Nagelkerke = 0.117; LRI = −18.758 (*p* = 0.085); the Hosmer–Lemeshow statistic = 8.528 (*p* = 0.384)					
Constant (y-intercept)	−5.029	2.501	4.145	0.006 (0.000–0.827)	0.042
TAS [mmol/L] in plasma	2.805	1.704	2.708	16.527 (0.585–466.664)	0.100

n—number of people; TAS—Total Antioxidant Status; LRI—the likelihood index chi-square test; b—regression coefficient in Wald-statistic; SE (b)—standard error in Wald-statistic; OR—Odds ratio; 95% CI—95% confidence interval; *p*—level of statistical significance.

## Data Availability

Data are contained within the article.
